# Operationalising kangaroo Mother care before stabilisation amongst low birth Weight Neonates in Africa (OMWaNA): protocol for a randomised controlled trial to examine mortality impact in Uganda

**DOI:** 10.1186/s13063-019-4044-6

**Published:** 2020-01-31

**Authors:** Melissa M. Medvedev, Victor Tumukunde, Ivan Mambule, Cally J. Tann, Peter Waiswa, Ruth R. Canter, Christian H. Hansen, Elizabeth Ekirapa-Kiracho, Kenneth Katumba, Catherine Pitt, Giulia Greco, Helen Brotherton, Diana Elbourne, Janet Seeley, Moffat Nyirenda, Elizabeth Allen, Joy E. Lawn

**Affiliations:** 1 Maternal, Adolescent, Reproductive, & Child Health Centre, London School of Hygiene & Tropical Medicine, Keppel Street, London, WC1E 7HT UK; 2 Department of Paediatrics, University of California San Francisco, 550 16th Street, Box 1224, San Francisco, CA 94158 USA; 3 Faculty of Epidemiology and Population Health, London School of Hygiene & Tropical Medicine, Keppel Street, London, WC1E 7HT UK; 4 Medical Research Council/Uganda Virus Research Institute and LSHTM Uganda Research Unit, PO Box 49, Entebbe, Uganda; 5 Department of Neonatal Medicine, University College London, 235 Euston Road, London, NW1 2BU UK; 6 Centre of Excellence for Maternal, Newborn, and Child Health, School of Public Health, Makerere University, New Mulago Hill Road, Kampala, Uganda; 7 Department of Public Health Sciences, Karolinska Institutet, SE-171 77 Stockholm, Sweden; 8 Faculty of Public Health and Policy, London School of Hygiene & Tropical Medicine, 15-17 Tavistock Place, London, WC1E 7HT UK; 9 Medical Research Council Unit The Gambia at LSHTM, PO Box 273, Fajara, The Gambia

**Keywords:** Preterm, Low birthweight, Newborn, Kangaroo care, Skin-to-skin contact, Neonatal mortality, Randomised controlled trial, Pragmatic

## Abstract

**Background:**

There are 2.5 million neonatal deaths each year; the majority occur within 48 h of birth, before stabilisation. Evidence from 11 trials shows that kangaroo mother care (KMC) significantly reduces mortality in stabilised neonates; however, data on its effect among neonates before stabilisation are lacking. The OMWaNA trial aims to determine the effect of initiating KMC before stabilisation on mortality within seven days relative to standard care. Secondary objectives include exploring pathways for the intervention’s effects and assessing incremental costs and cost-effectiveness between arms.

**Methods:**

We will conduct a four-centre, open-label, individually randomised, superiority trial in Uganda with two parallel groups: an intervention arm allocated to receive KMC and a control arm receiving standard care. We will enrol 2188 neonates (1094 per arm) for whom the indication for KMC is ‘uncertain’, defined as receiving ≥ 1 therapy (e.g. oxygen). Admitted singleton, twin and triplet neonates (triplet if demise before admission of ≥ 1 baby) weighing ≥ 700–≤ 2000 g and aged ≥ 1–< 48 h are eligible. Treatment allocation is random in a 1:1 ratio between groups, stratified by weight and recruitment site. The primary outcome is mortality within seven days. Secondary outcomes include mortality within 28 days, hypothermia prevalence at 24 h, time from randomisation to stabilisation or death, admission duration, time from randomisation to exclusive breastmilk feeding, readmission frequency, daily weight gain, infant–caregiver attachment and women’s wellbeing at 28 days. Primary analyses will be by intention-to-treat. Quantitative and qualitative data will be integrated in a process evaluation. Cost data will be collected and used in economic modelling.

**Discussion:**

The OMWaNA trial aims to assess the effectiveness of KMC in reducing mortality among neonates before stabilisation, a vulnerable population for whom its benefits are uncertain. The trial will improve understanding of pathways underlying the intervention’s effects and will be among the first to rigorously compare the incremental cost and cost-effectiveness of KMC relative to standard care. The findings are expected to have broad applicability to hospitals in sub-Saharan Africa and southern Asia, where three-quarters of global newborn deaths occur, as well as important policy and programme implications.

**Trial registration:**

ClinicalTrials.gov, NCT02811432. Registered on 23 June 2016.

## Background

An estimated 2.5 million neonatal deaths occurred in 2018, accounting for nearly half of all deaths in children aged < 5 years [1]. Within the neonatal period, 36% of deaths occur on the day of birth and 73% occur in the first week [2]. Over 80% of neonatal deaths occur in low birthweight (LBW; weighing < 2500 g) babies, of which two-thirds are born preterm (≤ 37 weeks gestational age) [3]. Complications of prematurity are the leading cause of neonatal and under-5 mortality [1]. Approximately two-thirds of the 21 million LBW and 15 million preterm babies born each year are born in sub-Saharan Africa or southern Asia [4–6]. Together, these two regions are responsible for 78% of neonatal deaths [1]. With rates of preterm birth rising or stagnant across the globe [5, 6], finding ways to improve survival and reduce morbidity in preterm babies is a growing imperative.

Substantial progress could be achieved by improving facility-based care of small and sick babies in low- and middle-income countries (LMIC) [7, 8], Estimates suggest that available interventions could reduce prematurity-related mortality by 58% [9]. Kangaroo mother care (KMC) is an intervention consisting of early skin-to-skin contact, promotion of exclusive breastmilk feeding, early hospital discharge, and adequate support and close follow-up at home [10]. The latest Cochrane review (21 trials) and a meta-analysis (124 studies) demonstrated that KMC among *stable* neonates ≤ 2000 g is associated with decreased mortality [11, 12], sepsis [11, 12], hypothermia [11, 12], hypoglycaemia [12] and length of stay [11] compared to conventional care. WHO guidelines recommend KMC for ‘routine care of newborns weighing ≤ 2000g… initiated as soon as newborns are clinically *stable*’ [13]; however, there is significant variability in how stability has been defined in previous randomised controlled trials (RCT) of KMC [14].

The majority of neonatal deaths occur within 48 h of birth [2], and before stabilisation. The only RCT of KMC initiated before stabilisation with mortality outcomes was conducted in Ethiopia, enrolling 123 newborns weighing < 2000 g [15]. It reported a 43% reduction in mortality; however, 66% of deaths and the major difference between arms occurred within 12 h of birth [15, 16]. Further, this trial excluded > 50% of eligible neonates, did not utilise allocation concealment and had an apparent group imbalance at baseline (favouring KMC) [15], compromising robustness. Hence, the effect of initiating KMC before stabilisation remains an unaddressed research priority and a well-designed RCT, with clear criteria for stability, is warranted to examine mortality impact in non-intensive care settings [16, 17]. The OMWaNA trial aims to determine the effect of KMC initiated before stabilisation on mortality within 7 and 28 days relative to standard care at four hospitals in Uganda.

There are few published economic evaluations of KMC, and none conducted rigorously in low-resource settings from a societal perspective or with systematic equity assessment. Several studies in LMIC settings have found that KMC resulted in cost savings for the hospital or provider [18–21]; however, none has considered whether KMC may increase costs to households nor purposely evaluated KMC initiated before stabilisation. Evidence gaps remain with regards to estimation of the incremental cost, cost-effectiveness, budget impact and equity of KMC before stabilisation, particularly considering the household and societal perspectives. An economic evaluation embedded within the trial will compare the incremental cost and cost-effectiveness of KMC relative to standard care.

Rigorous studies examining causal pathways for the effects of KMC on neonatal health outcomes have not been conducted; thus, scientific understanding is limited. Potential underlying mechanisms may include improved thermal control [11, 12], enhanced cardiorespiratory stability [22, 23], increased breastmilk volume [24, 25], oxytocin-mediated attenuation of the stress response [26, 27] and thermally mediated reduction in the risk of intraventricular haemorrhage (IVH) [28]. The relevance of these hypothesised causal pathways in neonates for whom KMC is initiated before stabilisation is unclear, particularly IVH risk among very low birthweight (VLBW; < 1500 g) newborns. Incubators, which are the standard alternative to KMC, may increase the risk of nosocomial infections, particularly in newborn units with ineffective cleaning standards or where incubators are shared [29, 30]. Thus, further research is warranted to improve scientific understanding of the physiological processes underlying the effect of KMC relative to standard care in this vulnerable population.

## Methods/design

This manuscript has been prepared according to the Standard Protocol Items: Recommendations for Interventional Trials (SPIRIT) statement (Additional file 1).

### Objectives

The primary objective of the OMWaNA trial is to determine the effect of KMC initiated before stabilisation on mortality within seven days relative to standard care among neonates weighing ≤ 2000 g. Secondary objectives include:

1. Determining the effect of KMC initiated before stabilisation on other important clinical outcomes relative to standard care among neonates weighing ≤ 2000 g;
2. Estimating the incremental costs and cost-effectiveness of KMC initiated before stabilisation relative to standard care from the societal perspective;
3. Exploring hypothesised causal pathways for the clinical effects of KMC initiated before stabilisation relative to standard care among neonates weighing ≤ 2000 g;
4. Examining the barriers and facilitators to initiating KMC before stabilisation to inform uptake and sustainability in Uganda.

### Study design

This is a four-centre, open-label, individually randomised, superiority trial with two parallel groups: an intervention arm allocated to receive KMC and a control arm allocated to receive standard care. Treatment allocation is random in a 1:1 ratio between groups.

### Study setting

**Fig. 1:**
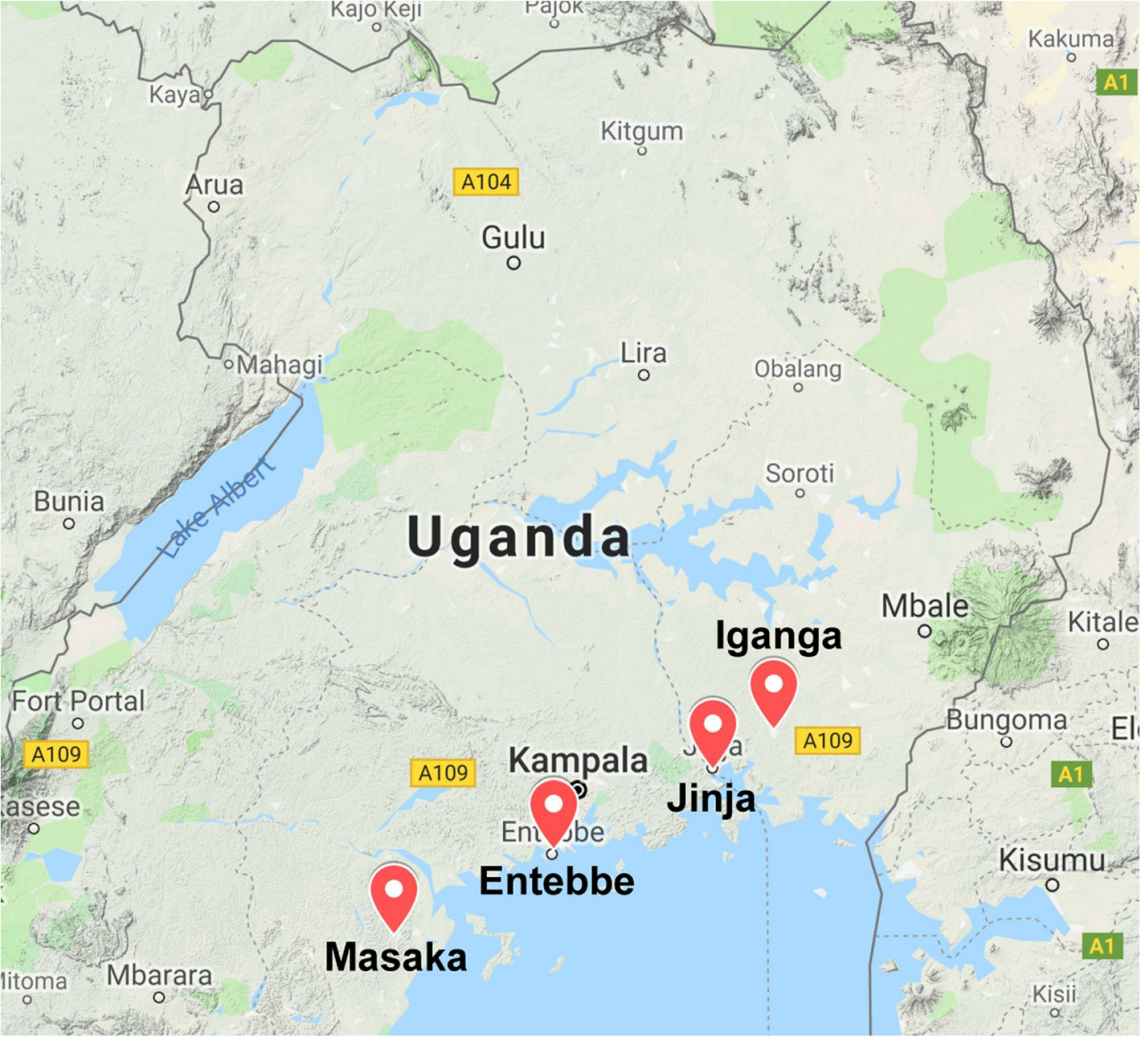
Map of Uganda showing location of the four OMWaNA trial hospitals. Source of map data: Google Maps©, 2019

The host institution for the trial is the Medical Research Council/Uganda Virus Research Institute (MRC/UVRI) and London School of Hygiene & Tropical Medicine (LSHTM) Uganda Research Unit in Entebbe. The trial is being undertaken in collaboration with Makerere University and LSHTM. The trial is being conducted at four Ugandan government hospitals: Entebbe, Jinja and Masaka Regional Referral Hospitals and Iganga District Hospital (Fig. 1).

Uganda has a population of 42.9 million and is ranked 162/189 on the Human Development Index (2017) [31]. The population is predominately rural (76%) and the poverty incidence is 27% nationally [32]. Poverty rates vary considerably, with the highest rates occurring in rural areas where subsistence farming is the primary source of income. The Busoga sub-region, where Jinja and Iganga are located, has the third highest incidence of poverty in the country (42.1%), while the Wakiso sub-region, where Entebbe is located, has the second lowest (7.5%) [32].

In Uganda, the neonatal mortality rate is estimated at 19.9 per 1000 live births, with a resultant 32,296 deaths in 2018 [1]. Complications of prematurity are responsible for 27% of neonatal deaths [33], as compared to 35% globally [1]. An estimated 107,921 (7%) Ugandan babies were born preterm in 2014 [5].

Characteristics of the four trial hospitals are shown in Table 1. Each hospital has a neonatal special care unit, which accepts referrals from their respective region/district. The level of equipment in these government facilities differs, but all have: bag-mask resuscitation; incubators and/or overhead radiant heaters for thermal support; intravenous (IV) fluids, nasogastric tubes and syringes for feeding support; oxygen supply (concentrators or cylinders) and nasal prongs for respiratory support; IV and oral antibiotics; phototherapy for jaundice; aminophylline for prematurity-associated apnoea; and phenobarbital for seizures. Pulse oximetry and improvised bubble continuous positive airway pressure (CPAP) ventilation are available at some of the trial hospitals. Invasive ventilation and surfactant are unavailable at all sites. Standard care at the four sites involves provision of intermittent KMC to neonates weighing ≤ 2000 g once stable, in line with current WHO guidelines.

**Table 1:** Characteristics of Ugandan trial hospitals, with resource availability in February 2019

	Entebbe Hospital	Iganga Hospital	Jinja Hospital	Masaka Hospital
Facility level of care	Regional	District	Regional	Regional
Catchment area [32]	Semi-urban	86% rural	86% rural	65% rural
Local poverty incidence (%) [32]	7.5	42.1	42.1	24.3
Live births (2018)	5706	6894	5287	9588
Neonatal admissions (2018)	597	933	698	2016
Born at an outside facility (n (%))	12 (2)	32 (3)	98 (14)	504 (25)
Birthweight < 2500 g (n (%))	248 (42)	421 (45)	234 (34)	NA^a^
Birthweight < 1500 g (n (%))	229 (38)	114 (12)	115 (17)	NA^a^
Average length of stay (days)	21	3–4	7	4
Paediatrician	1	1	3	2
Nurses in neonatal unit	8^b^	5	9	6
Overhead radiant heater	3 functional	1 functional	4 functional	2 functional
Incubator	2 functional	4 functional	3 functional, 6 non-functional	3 functional
Open cots	0	7	10	8
Oxygen supply	2 concentrators, 2 cylinders	1 concentrator, 1 cylinder	4 concentrators, 3 cylinders	2 non-functional concentrators, 1 cylinder
Bubble CPAP (improvised)	1	1	1	0
Pulse oximeter	0	1	4	1
Phototherapy	2 functional	1 functional, 1 non-functional	3 functional	4 functional
KMC beds, chairs	4 beds (KMC room), no chairs	5 beds (3 KMC room, 2 postnatal corner), no chairs	4 beds (KMC room), 20 chairs (neonatal unit)	4 beds (KMC room), no chairs

*CPAP* continuous positive airway pressure, *KMC* kangaroo mother care, *NA* not applicable

^a^ Neonatal admissions data were not available for Masaka Hospital

^b^ The neonatal unit at Entebbe Hospital has six government-employed nurses and two volunteer nurses

### Study population

The trial will include admitted neonates weighing ≤ 2000 g for whom the indication for KMC is ‘uncertain’ according to WHO guidelines concerning clinical stability [10]. Eligibility criteria are listed below.

*Inclusion criteria:*

- Neonate admitted to trial hospital (inborn or outborn);
- Singleton, twin or triplet (if triplet pregnancy resulted in demise or stillbirth of ≥ 1 fetus);
- Birthweight ≥ 700 g and ≤ 2000 g;
- Chronological age ≥ 1 h and < 48 h at time of screening;
- Alive at time of recruitment;
- Parent/caregiver able and willing to provide KMC;
- Parent/caregiver willing to attend follow-up visit;
- Indication for KMC ‘uncertain’ according to WHO guideline concerning clinical stability: pragmatically defined as receiving ≥ 1 therapy: oxygen; CPAP; IV fluids; therapeutic antibiotics (for suspected or confirmed infection); phenobarbital.

*Exclusion criteria:*

- Result of triplet or higher-order multifetal pregnancy (unless triplet pregnancy resulted in demise or stillbirth of ≥ 1 fetus);
- Indication for KMC ‘certain’ according to WHO guidelines: pragmatically defined as clinically well neonates receiving none of the above therapy-based criteria;
- Severely life-threatening instability defined as oxygen saturation (SpO_2_) < 88% in oxygen and ≥ 1 of:
  - Respiratory rate < 20 or > 100 breaths/min;
  - Apnoea requiring bag-mask ventilation;
  - Heart rate (HR) < 100 or > 200 beats/min;
- Severe jaundice requiring immediate management;
- Active neonatal seizures;
- Major congenital malformation;
- Parent does not provide written informed consent to participate in trial.

### Study procedures

**Fig. 2:**
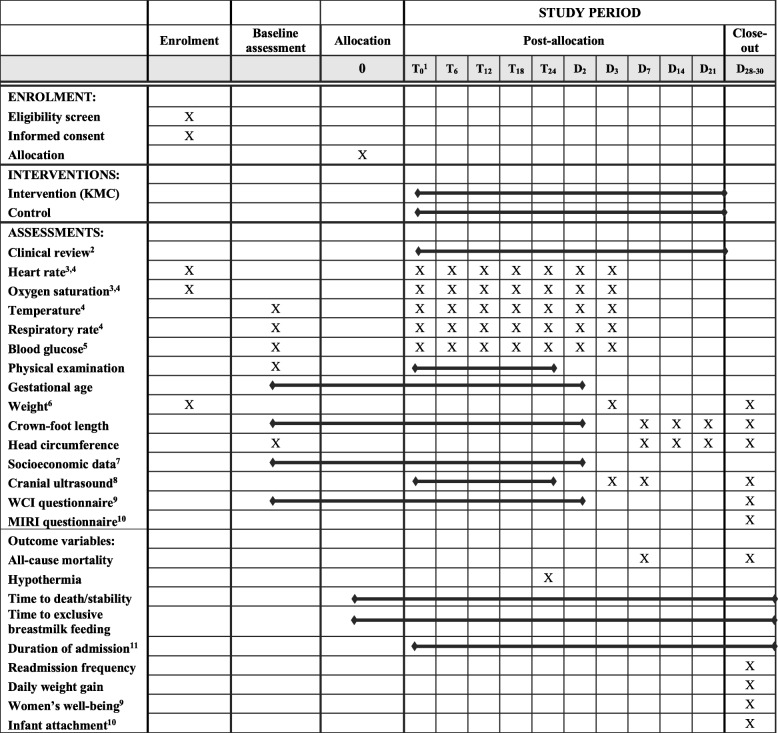
OMWaNA trial schedule of enrolment, interventions and assessments ^1^. The start of trial procedures (time 0) is defined as when the pulse oximeter is attached for cardio-respiratory monitoring ^2^. All participants are reviewed daily while admitted to the hospital ^3^. All participants receive continuous monitoring of heart rate (HR) and oxygen saturation (SpO_2_) for 72 h after randomisation. Continuous monitoring continues until participants no longer require any form of respiratory support ^4^. HR, SpO_2_, axillary temperature and respiratory rate are measured every 6 h until stability criteria are met, after which the frequency transitions to daily ^5^. Blood glucose is measured daily and may be discontinued once the participant tolerates full enteral feeds ^6^. Participants are weighed on day 5, then daily until discharge (unless deemed too unstable by site study staff) ^7^. Socioeconomic data, including household details, are collected within 48 h of enrolment. During this time, study staff also inform families that they will be asked about their household expenditures and activities over the coming month ^8^. For participants at Entebbe and Jinja Hospitals, cranial ultrasounds are performed on days 1, 3 and 7 of hospitalisation (or as an outpatient if discharged before day 7) and on follow-up at day 28–30 ^9^. The Women’s Capabilities Index (WCI) is administered to all mothers within 48 h of enrolment and on days 28–30 to assess women’s wellbeing ^10^. The Maternal Infant Responsiveness Instrument (MIRI) is administered on days 28–30 to assess infant-caregiver attachment ^11^. Duration of admission is measured as the mean time (days and hours) from hospital admission to discharge

The schedule of procedures for the OMWaNA trial is outlined in Fig. 2.

**Fig. 3:**
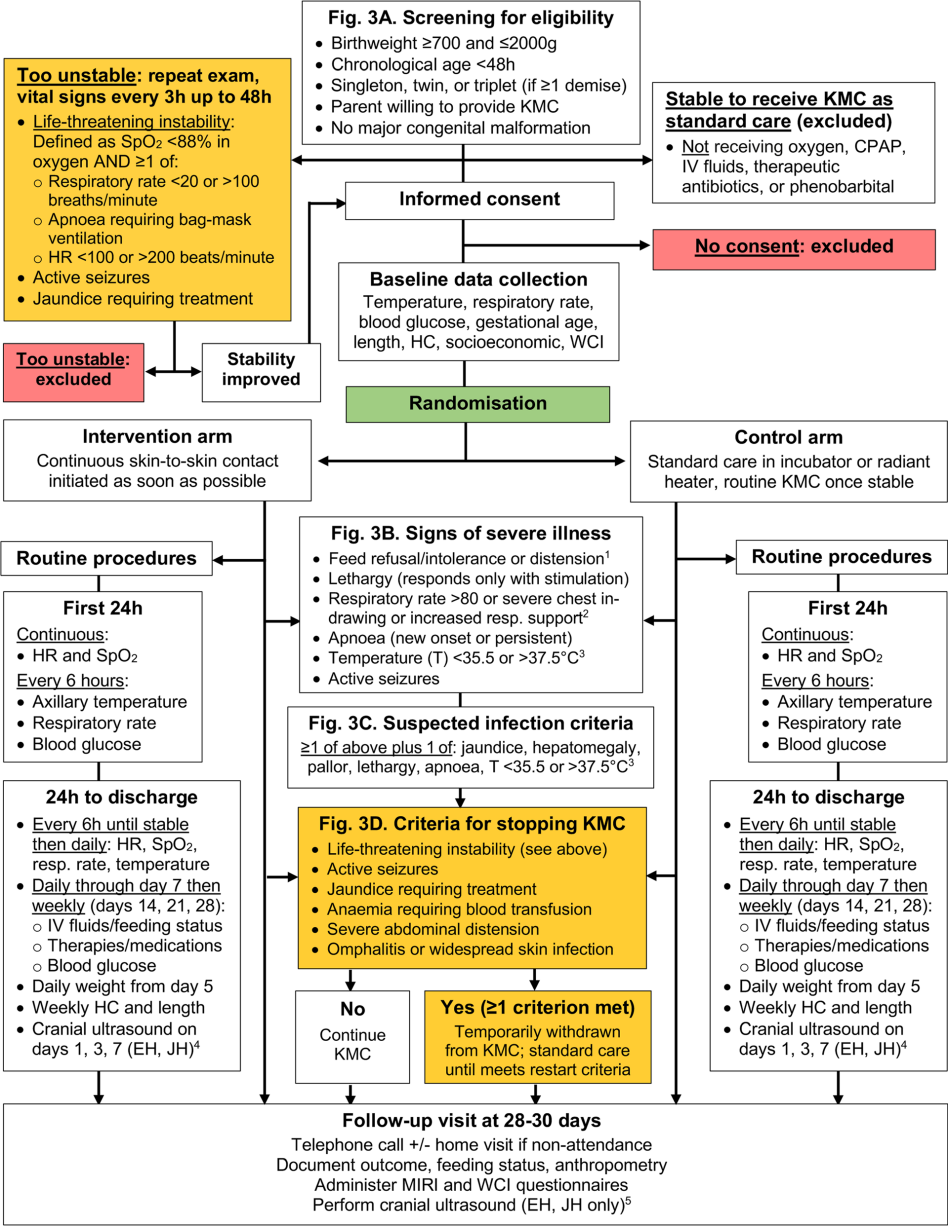
Overview of trial flow including routine procedures and key criteria for eligibility screening, assessing severe illness and stopping KMC ^1^. Refusal to feed, feed intolerance or abdominal distension (after starting feeds) ^2^. Increased respiratory support defined as new oxygen or CPAP requirement ^3^. Axillary temperature < 35.5°C after 1 h of observed skin-to-skin contact, not associated with environment or with hypoglycaemia ^4^. For participants at EH and JH, cranial ultrasounds will be performed on days 1, 3 and 7 of hospitalisation (or as an outpatient if discharged before day 7) and on follow-up at days 28–30. CPAP continuous positive airway pressure, EH Entebbe Hospital, HC head circumference, JH Jinja Hospital. **a** Screening for eligibility. **b** Signs of severe illness. **c** Suspected infection criteria. **d** Criteria for stopping KMC

Figure 3 describes the flow of participants from the time of screening through follow-up at 28–30 days.

### Screening

All admitted neonates weighing ≤ 2000 g at the four trial hospitals will be screened for eligibility by a study nurse or medical officer (Fig. 3a, ‘Screening for eligibility’). Eligibility will be assessed as soon as possible after admission and once the baby is aged ≥ 1 h to allow for transition immediately after birth. This is in recognition of the large physiological changes that take place following delivery and that the stability of a newborn aged < 1 h may change rapidly and not accurately reflect their subsequent clinical trajectory. Trained study staff will ascertain chronological age and relevant pregnancy details by examining source documents and/or conducting a standardised maternal interview. Weight will be measured using the Seca™ 384 electronic weighing scale. A focused examination will be conducted to assess for the presence of major congenital malformations, severe jaundice and seizures.

Neonates for whom KMC is indicated per WHO guidelines (i.e. are considered ‘stable’) will be excluded and receive KMC as part of standard care (Fig. 3a, ‘Stable to receive KMC’). Neonates for whom the indication for KMC is ‘uncertain’ per WHO guidelines (i.e. are ‘prior to stability’) will be further assessed. For those neonates who are found to meet eligibility criteria (Fig. 3a, ‘Screening for eligibility’), a trained member of the study staff will monitor HR and SpO_2_ using a Masimo Rad-8© pulse oximeter for 10 min and measure respiratory rate manually by counting breaths for 1 min. Those found to meet the criteria for ‘life-threatening instability’ (Fig. 3a, ‘Too unstable’), or who have seizures or jaundice requiring treatment, will not be eligible for immediate recruitment and will enter a cycle of reassessment every 3 h. All will continue to receive clinically indicated treatments and cardiorespiratory monitoring at the discretion of the on-duty paediatrician, medical officer or nurse. If, during any reassessment within the first 48 h, a neonate is found to have improved and no longer meets exclusion criteria, recruitment may proceed. Neonates who continue to have life-threatening instability or meet other exclusion criteria by 48 h will be permanently excluded.

### Informed consent

Written informed consent will be sought from the parents of all participants for the following: neonatal inclusion in the study; collection of sociodemographic and clinical data; and randomisation to a study arm. Consent will also be obtained for the possibility that the caregiver will provide continuous skin-to-skin contact, if randomised to that arm. Additionally, consent will be obtained for the collection of household socioeconomic and cost data, as well as data on infant–caregiver attachment and women’s wellbeing. Study medical officers or nurses will request informed consent. The preferred person to provide informed consent for neonatal involvement is the mother. If a mother is unavailable or too ill to provide consent, consent can be obtained from the father. Once the mother is available and feeling well enough, the informed consent process will be repeated to confirm her consent for her baby’s continued participation. An impartial and literate witness will be used during consent for non-literate parents, as per International Council for Harmonisation-Good Clinical Practice (GCP) guidance.

### Collection of baseline data

Study staff will be trained in infection prevention and standard operating procedures (SOP) will detail infection control measures for the use of study equipment to avoid contamination between participants. Axillary temperature will be measured with a digital thermometer in degrees Celsius; three measurements will be taken to enable calculation of the mean value. Respiratory rate will be measured manually by counting breaths for 1 min. Blood glucose will be measured with a capillary sample using the study glucometer. Head circumference (HC) will be measured and a physical examination will be conducted. Baseline clinical and anthropometric data will be collected as soon as possible after enrolment, with the exception of gestational age and crown-foot length, which may be delayed to within 48 h of enrolment. Gestational age will be estimated using Ballard score [34], last menstrual period and foot length [35]. Length will be measured using the Seca™ 210 neonatal measuring mat.

Socioeconomic data, including household details, will be collected within 48 h of enrolment using standardised parent interviews. The Women’s Capabilities Index (WCI) questionnaire will also be administered to mothers during this timeframe. Study staff will also inform families that, over the coming month, they will be asked about their expenditures and the activities of members of their household in order to evaluate the economic impact of KMC relative to standard care.

### Randomisation, allocation and blinding

Treatment allocation is random in a 1:1 ratio between groups using permuted blocks of varying block sizes. The allocation sequence was computer-generated centrally at MRC/UVRI by an independent statistician, stratified by birthweight (< 1000, 1000–1499 or ≥ 1500 g) and recruitment site. The random allocation sequence is uploaded onto the REDCap (Research Electronic Data Capture, Nashville, TN, USA) platform [36] and accessed using a computer with Internet access at each site. The randomisation server and research database are hosted at the MRC/UVRI and LSHTM Unit data centre. This precludes any possibility of study staff viewing the allocation sequence. Allocation is revealed only after the study medical officer or nurse has entered all required screening data into REDCap. The mother is the unit of randomisation; twin and triplet participants will be allocated to the same arm. Each site has one spare computer in case of breakdown or theft; if both fail, the site will revert to random allocation using telephone as the back-up option. Given the nature of the KMC intervention, blinding parents/caregivers is not possible. Process and outcome data will be anonymised and all analyses will be blinded. Analyses will be unblinded for the Data and Safety Monitoring Board (DSMB) at their request.

### Intervention arm

**Fig. 4:**
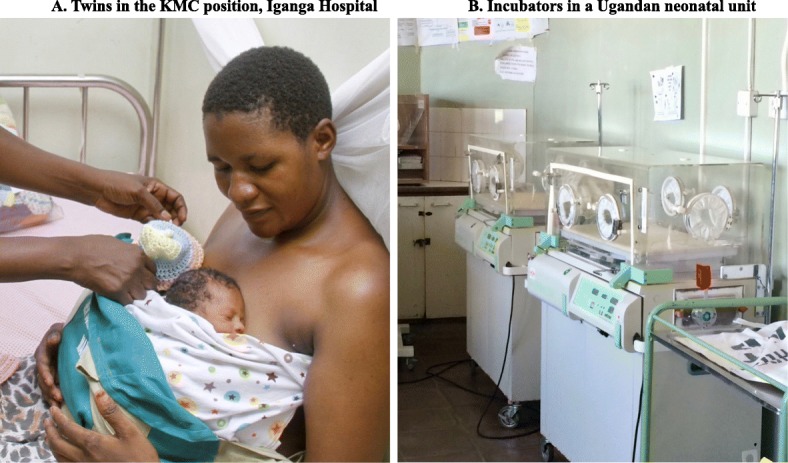
OMWaNA intervention (KMC) and control (standard incubator care) arms. Images: University of California San Francisco Preterm Birth Initiative, with caregiver consent for publication (**a**); Melissa Medvedev (**b**)

Neonates in the intervention group will receive KMC initiated as soon as possible after randomisation. Neonates will be naked except for hat and diaper, and will be secured to the exposed chest of the caregiver using a KMC wrap (Fig. 4a). The caregiver is seated or lying on a bed, while the neonate receives any clinically indicated therapies (e.g. IV fluids, antibiotics, oxygen). Caregivers will be encouraged to provide KMC as close to continuously as possible, aiming for at least 18 h per day. If the primary caregiver is unavailable, another family member (e.g. father, grandmother) or close friend (helper) will be encouraged to provide KMC. If a family member or helper is not available to continue KMC, the neonate will be placed into an incubator or under a radiant heater until the caregiver returns. KMC will continue to be encouraged until discharge and at home after discharge, as per WHO guidelines. KMC is commonly practiced until the baby is 2500 g or resists the KMC position, which is often at 4–10 weeks after birth.

Neonates who meet any of the criteria for stopping KMC (Fig. 3d, ‘Criteria for stopping KMC’) will be temporarily withdrawn from the intervention and cared for in an overhead heater or incubator at the discretion of the on-duty paediatrician. KMC may be restarted once all of the following criteria are met: (1) no longer meet any of the stopping criteria; (2) no apnoea requiring bag-mask ventilation for 24 h; (3) not on phototherapy; (4) no seizures for 24 h; (5) no abdominal distension; (6) caregiver available and willing to do KMC; (7) no healthcare worker concerns about clinical condition.

### Control arm

Neonates in the control group will be cared for in an incubator (Fig. 4b) or under a radiant heater. Caregivers are able to touch, hold and feed their baby, but may not provide any skin-to-skin contact until the neonate meets WHO criteria for KMC, i.e. are considered ‘stable’. Neonates will be considered stable when the following criteria have been met for a continuous period of ≥ 24 h: (1) breathing spontaneously with SpO_2_ > 90% in room air; (2) no need for supplemental oxygen or CPAP; (3) respiratory rate 40–< 60 breaths/min; (4) no apnoea; (5) HR 80–< 180 beats/min; (6) axillary temperature 36.0–37.4 °C; and (7) no need for IV fluids. These criteria are consistent with those being used in the WHO-led Immediate KMC (I-KMC) trial [37]. Once stable, neonates can transition to routine (intermittent) KMC with the caregiver in line with standard care at the trial sites. As in the intervention arm, neonates in the control arm who meet any of the criteria for stopping KMC (Fig. 3d, ‘Criteria for stopping KMC) will be cared for in an overhead heater or incubator until restart criteria are met.

### Participant flow around study sites

**Fig. 5:**
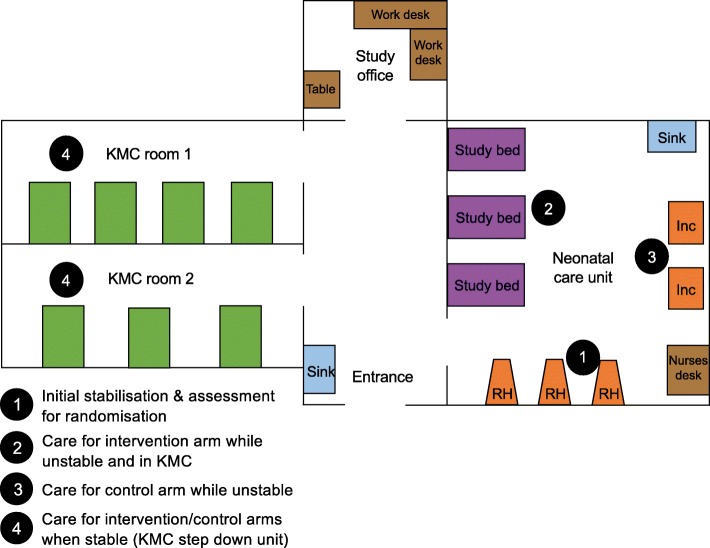
Study site participant flow for the OMWaNA trial. Inc incubator, RH radiant heater

Participant flow around the study sites is illustrated in Fig. 5, using Entebbe Hospital as an example. All neonates are initially stabilised and assessed at a radiant heater. While clinically ‘unstable’, neonates allocated to KMC are cared for in a study bed and those allocated to standard care are cared for in an incubator or radiant heater. Participants in both arms are transferred to the KMC step-down unit once they meet stability criteria.

### Neonatal care capacity building

Substantial expansion of neonatal care capacity and infrastructure at all trial sites has been embedded within the OMWaNA trial. This includes enlargement of the KMC areas within the neonatal units to ensure that all neonates, whether in KMC or not, can be cared for safely. Additional infrastructure improvements include sinks to provide an optimal environment for infection control, bathrooms/toilets for mothers/caregivers and office space for clinical staff. One study medical officer and 4–5 study nurses have been recruited to join the clinical teams at each site. Further, each site will be provided with the following supplies and equipment: six Masimo Rad-8© oximeters with neonatal sensors; one oxygen concentrator; two thermometers; one glucometer with blood glucose testing strips; one neonatal ventilation bag and mask; one Seca™ 384 neonatal weighing scale; one Seca™ 210 neonatal measuring mat; 2–3 paediatric stethoscopes; and a minimum of four adjustable beds. In addition, KMC wraps will be provided to support practice in each unit.

### Clinical care for neonates in both arms

#### Clinical monitoring

All participants will be evaluated at least once by a study paediatrician or medical officer during the first 24 h after randomisation. All participants will receive continuous monitoring and recording of HR and SpO_2_ for 72 h after randomisation. Continuous monitoring will continue until participants no longer require any form of respiratory support. HR, SpO_2_, axillary temperature and respiratory rate will be measured and recorded by a study nurse every 6 h until stability criteria are met, after which the frequency will transition to daily. According to the same frequency, a study nurse will observe and record the presence or absence of clinical signs of respiratory distress, including chest in-drawing, nasal flaring and grunting. Blood glucose will be measured every 6 h during the first 24 h after randomisation unless it is < 2.6 mmol/L, in which case it will be measured hourly until two or more consecutive readings are in normal range (2.6–6.9 mmol/L). Subsequently, blood glucose will be measured daily until a participant is tolerating full enteral feeds.

#### Medical therapies

All enrolled neonates will receive clinically indicated treatments, including but not limited to oxygen, IV fluids (given by bolus or burette), antibiotics, aminophylline, anticonvulsant medicines and phototherapy. Standardised clinical guidelines will be followed for common neonatal conditions, including preterm fluids/feeding (including breastfeeding), suspected and proven sepsis, respiratory distress, jaundice and seizures. Bubble CPAP will be provided at the discretion of the on-duty paediatrician at sites where this is the standard of care. Jaundice will be treated with phototherapy for neonates in both arms. All caregivers will be trained in KMC regardless of study arm.

#### Clinical deterioration

Neonatal unit staff at all sites will be trained to recognise signs of severe illness (Fig. 3b, ‘Signs of severe illness) and to inform study staff if a participant meets any of these criteria. The study paediatrician or medical officer (or study nurse if neither is present) will examine the neonate as soon as possible to assess whether signs of early-onset (< 72 h of age) or late-onset (≥ 72 h of age) infection (Fig. 3c, ‘Suspected infection criteria’) are present. Neonates will be reassessed for signs of severe illness and infection during daily rounds. Where available, a blood culture will be obtained as soon as possible if a neonate meets criteria for suspected infection; however, this will not delay administration of antibiotic therapy. Study staff will also assess if the neonate meets criteria for temporary withdrawal from KMC (Fig. 3d, ‘Criteria for stopping KMC’). At the discretion of the study paediatrician, neonates may be referred to a higher-level facility for more specialised care; however, existing data indicate that this is an uncommon occurrence.

### Discharge and follow-up

At the time of discharge, all caregivers will be provided with an illustrated handout on neonatal danger signs and instructed to contact the site study team or seek medical help if their baby becomes unwell. Caregivers of babies in both arms will be encouraged to continue KMC at home. All participants will be given an appointment to attend the follow-up clinic at the respective study site on days 28–30. At this visit, cranial ultrasound (Entebbe and Jinja Hospitals only) and anthropometry will be performed, feeding practices and outcomes (alive, dead, readmitted) will be documented, and the WCI and Maternal Infant Responsiveness Instrument (MIRI) questionnaires will be administered to mothers.

If participants are discharged before day 7, additional follow-up will be arranged according to study site. If participants do not attend the follow-up visit, a telephone call will be made the same day to ascertain outcome and feeding practices and to arrange follow-up, either in the clinic or at the families’ home, as soon as possible. Routine follow-up beyond the planned study follow-ups will be provided by the study staff according to standard practice and based upon the clinical need of the baby.

### Safety reporting and study monitoring

Adverse events (AE) are medical events or laboratory findings, which result in a change in clinical management after randomisation and until 28 days after birth. A serious adverse event (SAE) is defined as an event that results in death, is life-threatening, requires hospitalisation or prolongation of hospitalisation, results in persistent or significant disability, or requires intervention to prevent permanent impairment or damage [38]. Study medical officers and nurses will inform the site paediatrician about any SAE occurrence within 24 h. SAEs will be followed up by the paediatrician until their resolution or stabilisation, or until causality is determined to be unrelated to the trial intervention. If a serious *but unexpected* AE occurs, which might be related to the trial intervention, a SAE report will be submitted to the Research Ethics Committees (REC) at UVRI and LSHTM within 48 h of the investigators becoming aware of the event, with a follow-up report provided within a further five working days. This expedited reporting will be limited to those outcomes not already listed as primary or secondary outcomes, yet which might reasonably occur as a consequence of the trial intervention. All SAEs will be reported to the Sponsor and RECs as part of their respective annual progress and safety report.

The DSMB will oversee the overall integrity of the study, its safety and its continued relevance and ability to answer the primary objective. DSMB members include a perinatal epidemiologist/statistician (chair), a South African neonatologist and a neonatal bioethicist. The DSMB will receive a summary of SAEs after one month of recruitment, then move to every three or six months; the DSMB will decide the frequency following the first report. An interim analysis will be performed on the primary outcome when approximately half of neonates have been randomised. An independent statistician will perform the interim analysis, blinded to treatment allocation and report to the DSMB. Analyses will be unblinded at the request of the DSMB. In light of these data and other evidence from relevant studies, the DSMB will inform the Trial Steering Committee (TSC) if in their view: it is evident that no clear outcome will be obtained with the current trial design; they have a major ethical or safety concern; or it is evident that the intervention is clearly superior and continuing the trial would be unethical to those in the control arm. The TSC will make the final decision on study continuation.

The study will be monitored by the Reciprocal Monitoring Scheme of the East African Consortium for Clinical Research in collaboration with the Research Compliance and Quality Assurance section of the MRC/UVRI and LSHTM Unit. Dedicated study monitors, independent of the study team, will oversee progress and ensure the trial is conducted and data are handled in accordance with the protocol, SOPs and applicable ethical and regulatory requirements. In addition, the UVRI REC will conduct initial site visits with neonatal specialists from the Uganda Paediatrics Association and the Ugandan Ministry of Health Newborn Steering Committee. The trial may be subject to audit by LSHTM under their remit as Sponsor, the Study Coordination Centre and other regulatory bodies to ensure adherence to GCP.

### Outcome measures

The primary outcome is all-cause early neonatal mortality (within seven days). Estimates suggest that three-quarters of neonatal deaths occur in the first week of life [2].

Secondary outcomes are as listed below.

1. *Prevalence of hypothermia (axillary temperature < 36.5 °C) at 24 h after randomisation*
  Axillary temperature will be assessed using a digital thermometer.
2. *Time from randomisation to clinical stabilisation (days and hours)*
  The date and time of randomisation and clinical stabilisation will be prospectively recorded. Stability is defined as having met all of the following criteria for a continuous period of at least 24 h:
  Breathing spontaneously with SpO_2_ > 90% in room air;
  No need for supplemental oxygen or CPAP;
  Respiratory rate 40– < 60 breaths/min;
  No apnoea;
  HR 80– < 180 beats/min;
  Axillary temperature 36.0–37.4 °C;
  No need for IV fluids.
3. *Time from randomisation to death (days and hours)*
  The date and time of death will be prospectively recorded from the death certificate for in-hospital deaths. For deaths occurring after discharge, the date will be recorded according to verbal report by the parent/caregiver.
4. *Time from randomisation to exclusive breastmilk feeding (days and hours)*
  The date and time of randomisation and initiation of exclusive breastmilk feeding will be prospectively recorded. Exclusive breastmilk feeding is defined as receiving breastmilk, either directly from the breast or by nasogastric tube, bottle, cup or spoon after expression from the breast, as the sole source of nutrition [39].
5. *Mean duration of hospital admission (days and hours)*
  The date and time of admission and discharge will be documented prospectively for the first admission episode.
6. *All-cause mortality within 28 days*
  This outcome will be documented at the follow-up visit on days 28–30. If participants do not attend, a telephone call will be made the same day to ascertain outcome.
7. *Mean frequency of readmission at 28 days*
  Episodes in which a neonate who had been discharged from a hospital is readmitted to the same hospital during the first 28 days will be prospectively recorded. Episodes in which a neonate is readmitted to a different hospital will be recorded according to verbal report by the parent/caregiver on follow-up at days 28–30.
8. *Mean daily weight gain (g/day) at 28 days*
  Mean daily weight gain will be calculated as the difference between weight at enrolment and on follow-up at days 28–30, as measured by the study scale.
9. *Women’s wellbeing at 28 days*
  Women’s wellbeing will be assessed using the WCI, a capability-based composite measure of quality of life that will capture the broader effects to the mother of practicing KMC. The WCI includes six domains (physical strength, inner wellbeing, household wellbeing, community relations, economic security, happiness), with a total of 26 sub-dimensions [40]. Developed and validated in Malawi, the WCI was recently adapted for use in Uganda [41].
10. *Infant–caregiver attachment at 28 days*
  Infant–caregiver attachment will be assessed using the MIRI, a 22-item questionnaire that measures maternal recognition of responsiveness to infant cues, maternal recognition of infant responsiveness and difficulties in responsiveness [42]. The MIRI was developed and validated in the United States, and is now being used in Uganda [43].

### Process outcomes

Understanding the hypothesised causal pathways for clinical effects of the intervention (objective 3) will be achieved by measurement of the following process outcomes, which are categorised as providing very early (within 24 h), early (within 72 h) or late clinical impact.

1. *Cardiorespiratory stability within 24 h, 72 h after randomisation*
  Proportion of time spent with suboptimal HR (< 100 bpm) and SpO_2_ (< 85%) over the first 24 h and 72 h after randomisation, measured and recorded continuously using the study pulse oximeter.
2. *Prevalence of hypothermia (axillary temperature < 36.5 °C) at 24 h, 72 h after randomisation*
  Axillary temperature will be assessed using a digital thermometer.
3. *Hypothermia density within 24 h, 72 h after randomisation*
  Hypothermia density is defined as the proportion of time the axillary temperature is < 36.5 °C during a defined time period. Axillary temperature will be measured every 6 h during the first 24 h after randomisation and until clinically stable, after which it is measured daily.
4. *Prevalence of hypoglycaemia (blood glucose < 2.6 mmol/L) within 24 h, 72 h after randomisation*
  Blood glucose will be measured using a study glucometer and glucose testing strips.
5. *Presence and severity of IVH at 72 h, seven days after randomisation; presence of late intracerebral sequelae of prematurity at days 28–30*
  IVH is a complication of prematurity characterised by bleeding within the cerebral ventricles, typically originating from the periventricular germinal matrix; severity ranges from grade 1 (mild) to grade 4 (severe) [44]. Late intracerebral sequelae include cystic degeneration, post-haemorrhagic hydrocephalus and cerebral atrophy. The study paediatrician or medical officer at two of the four hospitals (Entebbe and Jinja) will perform cranial ultrasounds using a Sonosite Edge II© portable ultrasound machine. Both standard and linear probes will be used to assess for abnormalities according to a defined protocol and will include ≥ 11 coronal and sagittal views. Images will be read by an independent expert.

### Data collection, management and security

Trial data will be electronically entered into trial-specific case report forms on tablets using an offline, mobile REDCap application, with inbuilt ranges and consistency checks. Data from tablets will be synchronised once daily over a secure connection with the web-based REDCap database, hosted at the MRC/UVRI and LSHTM Unit data centre. Cardiorespiratory data from Masimo Rad-8© oximeters will be downloaded using Stowood Visi-Download™ software, captured in CSV files, securely transmitted to MRC/UVRI, analysed with PROFOX™ software and reconciled with the trial database. Cranial ultrasound images will be stored in OsiriX Dicom™ software and interpreted blind to allocation and clinical details. Logs linking parent/caregiver names and residence location will be stored separately on password-protected computers, with a hard copy stored in locked cabinets in secure rooms at all sites.

All data will be stored in institutional servers at the MRC/UVRI and LSHTM Unit during the study. Data from the web-based REDCap database will be downloaded and stored on institutional servers at LSHTM in London for access by the PIs and independent statistician for analysis and preparation of reports for the DSMB, respectively. These secure, password-protected servers are only accessible within the LSHTM network and activity is fully audited, recording both login details and file system access. Access will be limited to essential research personnel.

### Sample size

Assuming a control mortality rate of 25% across the four recruitment sites, 1750 neonates (875 per arm) would enable us to detect a relative difference between arms of 22.4% (5.6% absolute difference) with 80% power and a significance level of 5%. If the control mortality rate were in fact as low as 18%, we would still be able to detect a relative difference of 27% (absolute difference of 4.8%). We plan to recruit 2188 neonates (1094 per arm) in order to allow for 10% withdrawal due to clinical deteriorations and consent withdrawal, and 10% dilution due to non-compliance and loss to follow-up. This sample size would enable us to detect absolute reductions of 6.3% and 5.4% from control rates of 25% and 18%, respectively, with 90% power.

### Statistical analyses

#### Summary of baseline data and flow of patients

**Fig. 6:**
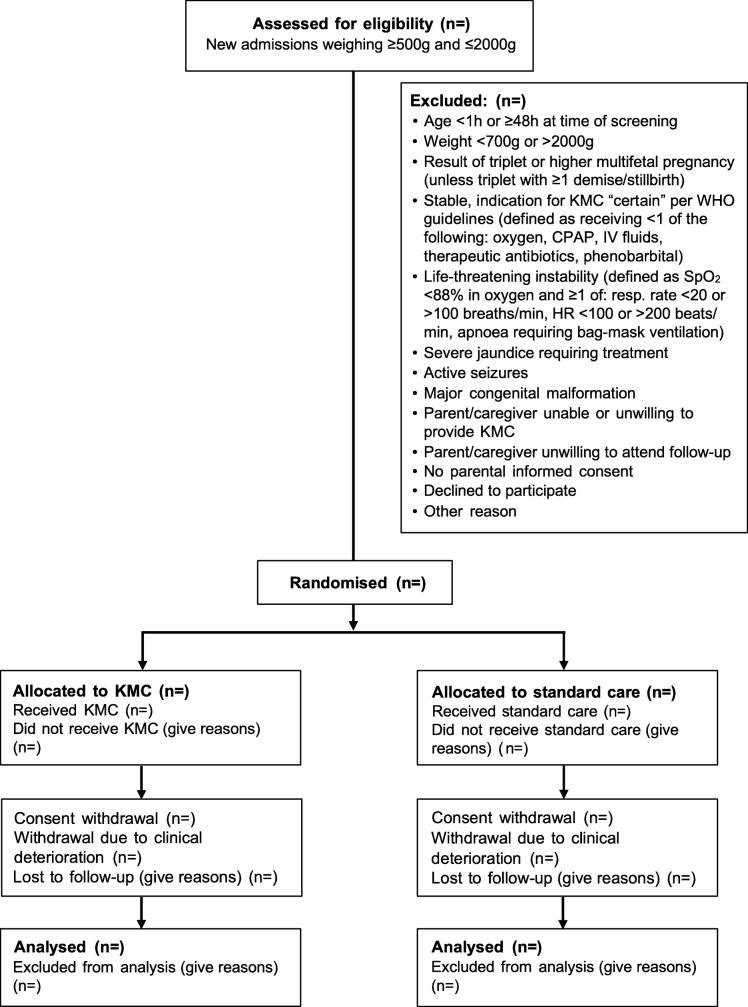
CONSORT flow diagram for the OMWaNA trial

Baseline characteristics of enrolled neonates will be summarised by treatment arm. Descriptive statistics for continuous variables will include mean, standard deviation, median, range and number of observations. Categorical variables will be summarised as counts and proportions. Participant flow through screening, randomisation, allocation and follow-up will be illustrated in a CONSORT diagram (Fig. 6), with reasons for exclusion, non-adherence, loss to follow-up and non-analysis documented.

#### Primary and secondary outcome analyses

Primary and secondary outcome analyses will be carried out on all neonates as randomised (‘intention-to-treat’). The rate of loss to follow-up will be reported. We will report risk ratios for mortality within seven days (primary outcome) and 28 days (secondary outcome) for intervention versus control with associated 95% confidence intervals (CI). Time from randomisation to death, time from randomisation to exclusive breastmilk feeding and length of stay will be analysed using Kaplan–Meier plots and hazard ratios, with accompanying 95% CI calculated using Cox proportional hazards regression. All other secondary outcomes will be analysed using appropriate regression models accounting for the nature of the distribution of the outcome, and results will be presented as appropriate effect sizes with a measure of precision (95% CI). Both unadjusted analyses and analyses adjusted for stratification factors will be carried out. Additional exploratory analyses will control for any baseline measures that appear to be imbalanced between arms.

#### Subgroup and adjusted analyses

Subgroup analyses are planned to explore between-group differences in the impact of KMC relative to standard care on mortality by gestational age (< 28, 28–32 or > 32 weeks), birthweight (< 1000, 1000–1499 or ≥ 1500 g) and recruitment site. Gestational age is an important predictor of newborn survival. In settings with newborn special care without intensive care, such as the four trial hospitals, neonatal mortality rates are 86% in neonates born at < 28 weeks and 41% in neonates born at 28–31 weeks [45]. Further exploratory analyses will be carried out to explore the association between mortality and time of initiation (< 12, 12– < 24 or ≥ 24 h), and continuity of KMC (median hours per day: < 6, 6– < 12, 12– < 18 or 18–24 h).

### Process evaluation

The process evaluation is being conducted to strengthen understanding of KMC initiation before stabilisation on neonatal health outcomes, considering both intended (beneficial) and unintended (negative) clinical effects. Changes in neonatal care between hospitals and from before the trial will also be assessed. This evaluation will be conducted in accordance with the MRC guidance on process evaluation of complex interventions [46], and will integrate quantitative and qualitative data. Quantitative outputs will include data related to causal pathways for clinical effects, neonatal admissions data, and health system- and facility-level survey data. Quantitative data will be summarised using descriptive statistics. Qualitative data will be collected though in-depth interviews, focus group discussions and workshops with parents/caregivers, healthcare providers and other key stakeholders to identify experiences of KMC and explore facilitators and barriers to inform uptake and sustainability. These data will be analysed using a thematic content approach. An iterative methodology will be used with data collected at several time points and then used to inform later explorations. Intervention reporting will follow the template for intervention description and replication (TIDieR) [47], which will ensure a shared understanding of all activities related to the trial intervention and, if shown to be effective, how these relate to any proposed scale-up activities. In addition, the TIDieR will facilitate thoughtful consideration regarding the transferability of findings outside a trial setting and to other hospitals in Uganda and elsewhere.

### Economic evaluation

The incremental cost, cost-effectiveness, budget impact and equity of KMC for neonates before stabilisation relative to standard care will be examined from both an aggregated and a disaggregated societal perspective (provider and household combined), in accordance with the reference case [48]. Effects of the intervention on neonatal health and maternal wellbeing will be assessed. Both financial costs, which reflect actual monies paid, and economic costs, which reflect the full value of resources used, will be examined. Multiple data sources will be triangulated to arrive at best estimates. Where possible, resource use and unit costs will be collected and presented separately, although some costs, such as out-of-pocket payments for transport, do not permit this. Household costs will be collected through surveys amongst a sample of caregivers at the time of discharge and during follow-up visits. Costs to providers will be collected prospectively and retrospectively using project accounts, key informant interviews, facility audits, direct observations and time-use surveys. As necessary, secondary data on costs of treating subsequent conditions may be supplemented with limited primary data collection in the trial hospitals. Costs and effects will be modelled using a lifetime time horizon.

## Discussion

Deaths in the neonatal period are responsible for 47% of mortality in children aged < 5 years [1]. Complications of prematurity are the leading cause, accounting for 35% of neonatal deaths and 16% of under-5 deaths [1]. The majority of neonatal deaths occur before stabilisation in settings without intensive care [2]. The OMWaNA trial will measure the impact of KMC initiated before stabilisation on mortality within seven days at four neonatal units in Uganda, where intensive care is not available. With rates of preterm birth and institutional delivery on the rise globally [5, 6, 49], this intervention has the potential to benefit an ever-growing number of neonates. This trial was designed with an aim to align clinical criteria and data definitions to facilitate future opportunities for pooled analyses with related RCTs, including the eKMC trial in The Gambia [50] and the multi-country I-KMC trial led by WHO [37]. Several challenges were identified over the course of designing the OMWaNA trial, the most notable of which are related to informed consent and recruitment, non-adherence and contextual resource limitations.

### Challenge 1: timely recruitment with informed consent

Obtaining timely informed consent for this RCT may be challenging given the involvement of sick neonates [51, 52], the fact that KMC needs to be started as soon as possible after birth and the fact that some women may be too ill, especially within the first 24 h, to provide consent or participate. In addition, some of these women may not be literate. Parental stress is compounded by the fact that complications may be unexpected, especially in low-resource settings, where knowledge of preterm birth is generally low. To help address these issues, the OMWaNA trial utilises the continuous consent approach [53], which involves providing information at multiple time points both before and after recruitment. Studies have found that the validity of consent improves when discussion continues after recruitment [53]. This approach has three main elements:

1. Parents will be given preliminary information during neonatal eligibility screening;
2. If the neonate is eligible, a comprehensive information sheet will be provided, and further discussion will take place. If the parents express willingness and ability to participate, written informed consent will be obtained and the neonate will be randomised;
3. During the intervention period, study staff will meet with parents to ensure that they understand the trial procedures and wish to continue to participate in the trial. It will be made clear that they may withdraw their baby from the trial at any time.

Audit data from the feasibility study at Jinja Hospital suggest that ~ 400 eligible neonates are admitted annually [14]. Preliminary data suggest that ~ 500 eligible neonates each are admitted to Iganga and Entebbe Hospitals per year and ~ 800 eligible neonates are admitted to Masaka Hospital per year. Thus, a total population of ~ 4400 eligible neonates is expected over the 24-month recruitment period, which means a recruitment rate of ~ 50% will be required to achieve the target sample of 2188. The feasibility study findings suggest that this is realistic and achievable [14]. The trial timeline includes a three-month buffer period in the event that recruitment is delayed or slower than expected. Further, training of study staff emphasised the importance of timely reporting and responsiveness to recruitment issues.

### Challenge 2: non-adherence to allocated treatment, especially continuous KMC

Among neonates in the control group, non-adherence with allocation (e.g. parents demanding early KMC) is a potential issue; however, this has not been reported in other trials [11]. Adherence in both arms, particularly the KMC arm, could be affected by parents witnessing a death (e.g. in the KMC position). Some babies will die regardless of the trial arm to which they are randomised. Preterm neonates can die quickly, even in settings with intensive care, and such deaths are a recognised impediment to KMC [54]. Stigma regarding preterm birth is common in Uganda, but has not impeded KMC practice for stable babies [55]. Study staff will counsel parents about the potential for mortality at the time of enrolment as well as counsel parents of neonates who die and those who witness a death. In addition, prompt reporting of all SAEs and trial monitoring through regular site visits will further facilitate timely identification of and responsiveness to any compliance issues, should they arise.

Adherence to the target duration of KMC (≥ 18 h/day) may be challenging [56]. Among five RCTs that promoted continuous KMC, three reported durations of ≥ 20 h/day [18, 57, 58] and two did not report duration [15, 59]. Among 16 RCTs evaluating intermittent KMC in stable neonates, one reported mean/median duration of 17 h/day, five reported 10–14 h/day and nine reported < 10 h/day [60]. The OMWaNA trial will employ a comprehensive approach to improve adherence. An illustrated KMC handout will be provided to caregivers at the time of enrolment. Study staff will counsel mothers about the benefits of KMC throughout the hospital stay, including the time of discharge. Studies have noted the importance of staff training and counselling for KMC [61, 62], and a related RCT demonstrated the efficacy of peer-counselling in promoting breastfeeding among hospitalised preterm neonates [63]. We will establish KMC peer-counselling programmes at each site, enlisting mothers who practiced KMC as participants when they return for follow-up. Peer counselling will address many maternal concerns and may help facilitate longer durations of KMC. We will also engage hospital administrators about KMC guidelines. Adjustable beds and KMC wraps will be provided, as these have been shown to improve adherence [56]. Lack of privacy and inadequate space for beds and equipment were identified as significant barriers to KMC practice in the feasibility study [14]. Provision of increased space within the four neonatal units may facilitate caregiver privacy as well as help improve clinical providers’ ability to safely care for at-risk neonates. Despite these measures, this is a pragmatic trial and some non-adherence is inevitable. The effect of non-adherence might be to dilute the size of the effect of KMC (assuming it ‘works’) and this has been factored into the sample size calculations. Biannual neonatal quality of care surveys, including progress monitoring of KMC provision/services, will be conducted at the four sites as part of the process evaluation.

### Challenge 3: context including infrastructure, supplies and equipment

Over the six months preceding the start of the trial, the study team has made extensive efforts to expand neonatal care capacity at the four hospitals. Improvements include increased space and water supply in the neonatal units, office space for clinical staff, bathrooms/toilets for caregivers, adjustable beds for KMC, and various equipment and supplies. Despite these efforts, context-related resource constraints are inevitable. In government facilities, such as the four trial hospitals, supply/medication shortages are common and repair of malfunctioning equipment (e.g. incubators, oxygen concentrators) is often protracted. Triannual (every four months) surveys of staffing, equipment, supply and medication availability, and infrastructure across the sites are included in the process evaluation. In addition to the supplies and equipment provided before trial commencement, the budget includes a small allowance to help cover the cost of necessary commodities for each participating neonate.

Electrical power is required for incubators, radiant warmers, oxygen concentrators and phototherapy. Lack of access to reliable electricity is a problem in many LMICs, including Uganda, where a 2007 national survey showed that only 19% of government hospitals had reliable electricity (during working hours) or a backup generator with fuel [64]. In 2014, a study at Jinja Hospital recorded 120 episodes of power failure (mean of 13 times/week), with a median duration of 30 min each, over a 64-day period [65]. The four trial hospitals have reliable power supply, with occasional brief power outages (e.g. when back-up generators run out of fuel). Daily communication between staff at the MRC/UVRI and LSHTM Unit and the sites will facilitate timely identification and resolution of any significant power outages impacting patient care. Frequency and duration of power outages will be recorded by site staff.

## Conclusion

The OMWaNA trial will assess the effectiveness of KMC in reducing mortality among neonates before stabilisation, a population where the benefits of KMC are currently uncertain. These findings are expected to have broad applicability to hospitals in low-resource settings and important policy and programme implications. The trial will be among the first to rigorously compare the incremental cost and cost-effectiveness of KMC and standard care, taking into account family members’ time, which will be crucial to ensure the sustainability of this intervention. Additionally, OMWaNA will advance understanding of the underlying mechanisms for the effects of KMC before stabilisation, including prematurity-associated brain injury, which could help inform prevention of disability as well as guide further innovation to improve survival for preterm newborns in the highest mortality settings.

## Supplementary information

**Additional file 1.** SPIRIT checklist.

**Additional file 2.** LSHTM ethics letter.

**Additional file 3.** UVRI ethics letter.

**Additional file 4.** UNCST ethics letter.

**Additional file 5.** Funding documentation.

## Data Availability

The final trial dataset will be available upon request to the PI or an institutional delegate. We plan to publish the results of the trial and the economic and process evaluations in peer-reviewed journals in an open access format. Manuscripts resulting from the trial will adhere to the Consort Guidelines and to authorship criteria set by the International Committee of Medical Journal Editors. We plan to present study results at international meetings and communicate findings to key audiences, including the WHO and the Ugandan Ministry of Health. Findings will be shared with relevant local stakeholders and participants’ families, adapted for those affected by a death.

## Abbreviations

AE: Adverse event
CI: Confidence interval
CONSORT: Consolidated Standards of Reporting Trials
CPAP: Continuous positive airway pressure
DSMB: Data and Safety Monitoring Board
EH: Entebbe Hospital
GCP: Good Clinical Practice
HC: Head circumference
HR: Heart rate
IV: Intravenous
IVH: Intraventricular haemorrhage
JH: Jinja Hospital
KMC: Kangaroo mother care
LBW: Low birthweight
LMIC: Low- and middle-income countries
LSHTM: London School of Hygiene & Tropical Medicine
MIRI: Maternal Infant Responsiveness Instrument
MRC: Medical Research Council
OMWaNA: Operationalising kangaroo Mother care before stabilisation amongst low birth Weight Neonates in Africa
PI: Principal investigator
RCT: Randomised controlled trial
REC: Research Ethics Committee
SAE: Serious adverse event
SOP: Standard operating procedure
SPIRIT: Standard Protocol Items: Recommendations for Interventional Trials
SpO_2_: Oxygen saturation measured by pulse oximetry
TSC: Trial Steering Committee
UVRI: Uganda Virus Research Institute
WCI: Women’s Capabilities Index
WHO: World Health Organization
