# Supplementary material for: Operationalising kangaroo Mother care before stabilisation amongst low birth Weight Neonates in Africa (OMWaNA): protocol for a randomised controlled trial to examine mortality impact in Uganda
Source: Trials. 2020 Jan 31;21:126. doi: 10.1186/s13063-019-4044-6 (PMC6995072; doi:10.1186/s13063-019-4044-6)
Supplement: Supplementary file 3 — Additional file 3. UVRI ethics letter. [file 13063_2019_4044_MOESM3_ESM.pdf]

UNHRO
Uganda National Health Research Organisation

**Uganda Virus Research Institute**
Plot 51-59, Nakiwogo Road, Entebbe
P.O. Box 49, Entebbe-Uganda
Tel: +256 414 320 385 / 6
Fax: +256 414 320 483
Email: directoruvri@uvri.go.ug

REPUBLIC OF UGANDA

Our Ref: GC/127/19/06/717

Your Ref:

## UVRI REC APPROVAL NOTICE

**To:** Dr. Cally Tann, Principal Investigator

**Re:** Application Tittle: **"The OMWANA study: Operationalizing kangaroo mother care before stabilization among low birth weight Neonates in Africa: a multi-site randomized controlled trial to examine mortality impact in Uganda."**

**Type:** [✓] Initial Review

I am pleased to inform you that REC meeting 04 convened on May 30, 2019 the UVRI REC voted to approve the above referenced application.

Approval of research is for the period of June 12, 2019 to June 12, 2020.

This research is for considered minimal risk for pediatric risk category. [✓] check box if not applicable

As principal investigator of the research, you are responsible for fulfilling the following requirements of approval:

1. All co-investigators of the research must be kept informed of the status of research.
2. Changes, amendments and addenda to protocol or the consent form must be submitted to the REC for review and approval **prior** to activation of changes. The REC application number assigned to the research should be cited in any correspondence.
3. Reports of unanticipated problems involving risks to participants or other must be submitted to the REC. New information that becomes available which could change the risk: benefit ratio must be submitted to REC.
4. Only approved consent forms are to be used in enrollment of participants. All consent forms signed by subjects and/or witness should be retained on file. The REC may conduct audits of all study records, and consent documentation may be part of such audits.
5. Regulations require review of an approved study not less than once per 12-month period. **Therefore, a continuing review application must be submitted to REC eight weeks** prior to the **above expiration date of** June 12, 2020 **in order to continue with the study beyond the approved period.** Failure to submit a continuing review application in timely fashion may result in suspension of the study. At which point new participants may not be enrolled and currently enrolled participants must be taken off the study.

UGANDA VIRUS RESEARCH INSTITUTE ENTEBBE
RESEARCH ETHICS COMMITTEE
APPROVED
VALID UNTIL
12 JUN 2020
P. O. BOX 49, ENTEBBE
UGANDA

6. You are required to register the research protocol with the Uganda National Council of Science and Technology (UNCST) for final clearance to undertake the study in Uganda.

**NOTE:** This study, being considered high risk will be closely monitored by the REC.

The following is the list of all documents approved in this application by UVRI REC:

| Document Tittle | Language | Version | Date |
|---|---|---|---|
| OMWaNA protocol | English | 1.1 | 17th May 2019 |
| OMWaNA ICF (English, Luganda and Lusoga ) | English, Luganda & Lusoga. | 1.0 | 8th February 2019 |
| OMWaNA ICF Photography and medical images (English, Luganda and Lusoga ) | English, Luganda & Lusoga. | 1.0 | 8th February 2019 |
| OMWaNA ICF Qualitative ( English, Luganda and Lusoga ) | English, Luganda & Lusoga. | 1.0 | 8th February 2019 |

Yours sincerely,

Dr. Tom Lutalo
**Chair, UVRI REC**
C.C Secretary, UVRI REC

UGANDA VIRUS RESEARCH INSTITUTE ENTEBBE
RESEARCH ETHICS COMMITTEE
APPROVED
12 JUN 2020
P. O. BOX 49, ENTEBBE
UGANDA
