# Supplementary material for: Operationalising kangaroo Mother care before stabilisation amongst low birth Weight Neonates in Africa (OMWaNA): protocol for a randomised controlled trial to examine mortality impact in Uganda
Source: Trials. 2020 Jan 31;21:126. doi: 10.1186/s13063-019-4044-6 (PMC6995072; doi:10.1186/s13063-019-4044-6)
Supplement: Supplementary file 5 — Additional file 5. Funding documentation. [file 13063_2019_4044_MOESM5_ESM.pdf]

MRC Medical Research Council

Head of Department
Research Contracts
London School Hygiene & Trop Medicine
Kepple Street
London United Kingdom
WC1E 7HT

Grant Ref: MR/S004971/1

Date: 29 November 2018

Dear Head of Department

GRANT OFFER: Research Grant, Joint Funded Initiatives Full
GRANT TITLE: OMWaNA Operationalising kangaroo Mother care before stabilisation among low birth Weight Neonates in Africa: RCT to examine mortality impact in Uganda

The MRC is offering a grant towards the cost of the above project, subject to the terms and conditions set out below.

Return of the 'Offer Acceptance' will be taken as acceptance of the grant on the terms stated. If you are unable to accept the grant you should return a 'Decline' confirmation as soon as possible. Upon receipt of the 'Offer Acceptance' a 'Start Confirmation' request will be issued.

Grants are cash limited and expenditure against the grant must not exceed the value awarded apart for reasons stated in the standard terms and conditions.

Please note copies of this letter have not been sent to the grant holder and co-investigators (as appropriate); it is your responsibility to distribute copies as is necessary.

Yours faithfully

Grants Pre Award Team
RCUK Grants
A service provided on behalf of MRC

Organisation: London Sch of Hygiene and Trop Medicine

Grant Holder: Professor Joy Lawn

Grant Title: OMWaNA Operationalising kangaroo Mother care before stabilisation among low birth Weight Neonates in Africa: RCT to examine mortality impact in Uganda
Starts: 1 December 2018 **Ends:** 31 May 2022 **Duration:** 42

GRANT VALUE

Funds Awarded

	Authorised FEC (£)			RC Contribution (£)			
	net	Indexation	Total	net	Indexation	Total	% FEC
DI - Staff	466,924	8,002	474,927	345,524	5,922	351,446	74
DI - T&S	59,509	1,110	60,619	44,037	821	44,858	74
DI - Equipment	86,465	0	86,465	86,465	0	86,465	100
DI - Other Costs	158,855	2,962	161,817	117,552	2,192	119,744	74
DA - Investigators	126,686	2,362	129,048	93,748	1,748	95,496	74
DA - Estate Costs	86,459	1,612	88,071	63,980	1,193	65,173	74
Indirect - Indirect Costs	481,337	8,975	490,312	356,189	6,641	362,831	74
Exception - Staff	60,090	979	61,069	60,090	979	61,069	100
Exception - Other Costs	744,813	13,888	758,701	744,813	13,888	758,701	100
**Total Value of Award**	**2,271,138**	**39,890**	**2,311,028**	**1,912,398**	**33,384**	**1,945,781**	
