# Supplementary material for: Operationalising kangaroo Mother care before stabilisation amongst low birth Weight Neonates in Africa (OMWaNA): protocol for a randomised controlled trial to examine mortality impact in Uganda
Source: Trials. 2020 Jan 31;21:126. doi: 10.1186/s13063-019-4044-6 (PMC6995072; doi:10.1186/s13063-019-4044-6)
Supplement: Supplementary file 2 — Additional file 2. LSHTM ethics letter. [file 13063_2019_4044_MOESM2_ESM.pdf]

London School of Hygiene & Tropical Medicine
Keppel Street, London WC1E 7HT
United Kingdom
Switchboard: +44 (0)20 7636 8636

www.lshtm.ac.uk

Observational / Interventions Research Ethics Committee

Prof Joy Lawn
LSHTM

11 July 2019

Dear Prof Joy Lawn ,

Study Title: The OMWaNA Study: Operationalising kangaroo Mother care before stabilisation amongst low birth Weight Neonates in Africa: a multi-site randomised controlled trial to examine mortality impact in Uganda

LSHTM ethics ref: 16972

Thank you for your application for the above research, which has now been considered by the Interventions Committee.

Confirmation of ethical opinion

On behalf of the Committee, I am pleased to confirm a favourable ethical opinion for the above research on the basis described in the application form, protocol and supporting documentation, subject to the conditions specified below.

Conditions of the favourable opinion

Approval is dependent on local ethical approval having been received, where relevant.

Approved documents

The final list of documents reviewed and approved by the Committee is as follows:

Document Type	File Name	Date	Version
Other	GCP_GCP Certificate (R2)_CT_12Dec17	12/11/2017	n/a
Other	RETC_Certificate_2019	09/01/2019	n/a
Information Sheet	ICFphotography_OMWaNA_V1.0_8.2.19	08/02/2019	1.0
Information Sheet	ICFquali_OMWaNA_V1.0_English_8.02.19	08/02/2019	1.0
Other	Melissa Medvedev_GCP Certificate_5May2019	05/05/2019	n/a
Investigator CV	Cally Tann_CV	30/05/2019	n/a
Investigator CV	Melissa Medvedev_CV	30/05/2019	n/a
Investigator CV	Peter Waiswa_CV	30/05/2019	n/a
Investigator CV	Joy Lawn_CV	30/05/2019	n/a
Investigator CV	Liz Allen_CV	30/05/2019	n/a
Sponsor Letter	2019-MUU-234_Sponsor Confirmation	31/05/2019	n/a
Protocol / Proposal	OMWaNA protocol_V1.2_31.05.19_clean	31/05/2019	1.2
Information Sheet	ICF_OMWaNA_V1.1_English_31.05.19_clean	31/05/2019	1.1

After ethical review

The Chief Investigator (CI) or delegate is responsible for informing the ethics committee of any subsequent changes to the application. These must be submitted to the Committee for review using an Amendment form. Amendments must not be initiated before receipt of written favourable opinion from the committee.

The CI or delegate is also required to notify the ethics committee of any protocol violations and/or Suspected Unexpected Serious Adverse Reactions (SUSARs) which occur during the project by submitting a Serious Adverse Event form.

An annual report should be submitted to the committee using an Annual Report form on the anniversary of the approval of the study during the lifetime of the study.

At the end of the study, the CI or delegate must notify the committee using an End of Study form.

All aforementioned forms are available on the ethics online applications website and can only be submitted to the committee via the website at: http://leo.lshtm.ac.uk

Additional information is available at: www.lshtm.ac.uk/ethics

Yours sincerely,

Professor Jimmy Whitworth
Chair

ethics@lshtm.ac.uk
http://www.lshtm.ac.uk/ethics/
