# Supplementary material for: Operationalising kangaroo Mother care before stabilisation amongst low birth Weight Neonates in Africa (OMWaNA): protocol for a randomised controlled trial to examine mortality impact in Uganda
Source: Trials. 2020 Jan 31;21:126. doi: 10.1186/s13063-019-4044-6 (PMC6995072; doi:10.1186/s13063-019-4044-6)
Supplement: Supplementary file 4 — Additional file 4. UNCST ethics letter. [file 13063_2019_4044_MOESM4_ESM.pdf]

Uganda National Council for Science and Technology

(Established by Act of Parliament of the Republic of Uganda)

UNCST

Our Ref: HS 2645 **30th August 2019**

Prof. Joy Elizabeth Lawn
Principal Investigator
C/o MRC/UVRI & LSHTM Research Unit
Entebbe

Dear Prof. Lawn,

Re: Research Approval: The OMWaNA Study: Operationalizing Kangaroo Mother Care before Stabilization amongst Low Birth Weight Neonates in Africa: A Multi – Site Randomized Controlled Trial to Examine Mortality Impact in Uganda

I am pleased to inform you that on **20/08/2019**, the Uganda National Council for Science and Technology (UNCST) approved the above referenced research project. The Approval of the research project is for the period of **20/08/2019** to **20/08/2022.**

Your research registration number with the UNCST is **HS 2645.** Please, cite this number in all your future correspondences with UNCST in respect of the above research project.

As Principal Investigator of the research project, you are responsible for fulfilling the following requirements of approval:

1. All co-investigators must be kept informed of the status of the research.
2. Changes, amendments, and addenda to the research protocol or the consent form (where applicable) must be submitted to the designated Research Ethics Committee (REC) or Lead Agency for re-review and approval **prior** to the activation of the changes. UNCST must be notified of the approved changes within five working days.
3. For clinical trials, all serious adverse events must be reported promptly to the designated local IRC for review with copies to the National Drug Authority.
4. Unanticipated problems involving risks to research subjects/participants or other must be reported promptly to the UNCST. New information that becomes available which could change the risk/benefit ratio must be submitted promptly for UNCST notification after review by the REC.
5. Only approved study procedures are to be implemented. The UNCST may conduct impromptu audits of all study records.

LOCATION/CORRESPONDENCE

Plot 6 Kimera Road, Ntinda
P.O.Box 6884
KAMPALA, UGANDA

COMMUNICATION

TEL: (256) 414 705500
FAX: (256) 414-234579
EMAIL: info@uncst.go.ug
WEBSITE: http://www.uncst.go.ug

6. An annual progress report and approval letter of continuation from the REC must be submitted electronically to UNCST. Failure to do so may result in termination of the research project.

Below is a list of documents approved with this application:

	Document Title	Language	Version	Version Date
1.	Research proposal	English	1.1	May 2019
2.	Informed Consent Form (ICF): The OMWaNA study	English, Luganda and Lusoga	1.0	February 2019
3.	ICF: Photography and medical images	English, Luganda and Lusoga	1.0	February 2019

Yours sincerely,

Isaac Makhuwa
For: Executive Secretary
UGANDA NATIONAL COUNCIL FOR SCIENCE AND TECHNOLOGY

Copied to: Chair, Uganda Virus Research Institute, Research Ethics Committee
